# Spectral Signatures of Immature *Lucilia sericata* (Meigen) (Diptera: Calliphoridae)

**DOI:** 10.3390/insects8020034

**Published:** 2017-03-23

**Authors:** Jodie-A. Warren, T. D. Pulindu Ratnasekera, David A. Campbell, Gail S. Anderson

**Affiliations:** 1 School of Criminology, Simon Fraser University, Burnaby, BC V5A 1S6, Canada; ganderso@sfu.ca; 2 Department of Statistics and Actuarial Sciences, Simon Fraser University, Burnaby, BC V5A 1S6, Canada; pratnase@sfu.ca (T.D.P.R.); dac5@sfu.ca (D.A.C.)

**Keywords:** *Lucilia sericata*, hyperspectral, remote sensing, reflectance, wavelength, functional regression, coefficient

## Abstract

Hyperspectral remote sensing is an innovative technology with applications in many sciences and is a non-destructive method that may offer more precise aging within development stages. Hyperspectral reflectance measurements from the anterior, midsection, and posterior of *Lucilia sericata* (Meigen) larvae and pupae were conducted daily from samples of the developing insects beginning at second instar. Only midsection measurements were conducted on second instar larvae due to their size, to ensure that the measurement was not of reflective surroundings. Once measured, all insects were washed with deionized water, blotted with filter paper, and re-measured. Daily age prediction during the post-feeding stage was not impacted by the unwashed insect measurements and was best predicted based on posterior measurements. The second and third instar larvae, which move about their food source, had different contributing coefficients to the functional regression model for the hyperspectral measurements of the washed compared with unwashed specimens. Although washing did not affect the daily prediction within these stages, it is still encouraged in order to decrease the effect of food source on spectral reflectance. Days within the intra-puparial period were best predicted based on anterior measurements and were not well distinguished from one another in the first few days based on midsection and posterior measurements.

## 1. Introduction

Hyperspectral remote sensing is an innovative geographic technique that applies the science of the electromagnetic spectrum, and has been used in many other sciences including many of the forensic sciences. Remote sensors measure reflectance, transmittance, emittance, and absorbance of radiation from a target surface [1]. Hyperspectral remote sensing is not invasive, is non-destructive to the sample, and can be used to identify surface features to light penetrable depths and express them as spectral signatures [1,2].

Hyperspectral remote sensing has been used in entomology to monitor insect damaged crops and the crop damaging species themselves [3,4,5,6,7,8,9,10,11,12,13,14], as well as to monitor insect vectors carrying diseases [15,16]. Besides monitoring entire population clusters, the non-invasive methods have been implemented in examining stress responses in single adult beetles exposed to killing agents [1] and used on insect eggs to determine whether they have been parasitized by *Trichogramma* wasps [17].

Forensic entomologists provide minimum post-mortem intervals (minPMI) based on the stage reached by the oldest insects developing on the remains. Forensic entomologists provide accurate minimum estimates of insect age, but seek more precision in these estimates [18]. Although a well-established science, forensic entomology, particularly the assessment of blow fly immature development, does have one major shortcoming and that is estimating time within stadia. Current means provide the estimated time it takes to reach a stage from the time of colonization and offer that to death investigators as an estimated minPMI [19,20]. This estimation is far from wrong, but is an underestimate, and this is compounded in longer duration stadia. Ideally, precision would be improved if forensic entomologists could provide a minimum estimate within the stage itself. Development demarcations within stages, particularly the latter stages, could condense the minimum estimate of tenure on the remains and hence provide more precision to the post-mortem interval [21].

There have been many efforts to solve this problem. Several methods of aging immature Calliphoridae are currently being applied by forensic entomologists, but these methods can be complicated by several limitations. As already stated, the most conservative method is to examine the stage reached and to provide an estimated age based on the length of time required to reach that stage. The estimated time to reach each stage is based on development data collected from a similar geographic area and temperature. The greatest limitation with this method is that a minPMI is provided based on the time required to reach the beginning of the stage for the oldest species at that temperature. Unfortunately, the latter stages of the life cycle can be quite lengthy, and so the estimated minPMI is a cautious estimate and lacks precision. Hyperspectral remote sensing can be used to measure reflectance of light from the surface of an insect at many wavelengths, and these measurements can be compared over time such that minute surface changes can be detected. The application of hyperspectral remote sensing to immature blow fly development can provide more precision to forensic entomology by aging within immature stage, rather than to a developing stage, and is not invasive or destructive to the insect [20].

Similarly, another common method is to apply the thermal summation required to reach each of these stages. This is done by examining the required accumulated degree days or hours (ADD/H) and applying this to the stage of the oldest insects on the remains. This method, too, provides an estimate to reach each stage and suffers the same limitation during lengthy stages. A second limitation is that this method is best used only when the ADD/H used to estimate the minPMI were generated at a nearby temperature [22,23].

Measurements of weight, length, and width of developing larvae are being used to estimate the minPMI [24,25,26]. The drawback to these methods is that carrion is an ephemeral food source and if the food source becomes depleted the larvae will be smaller in size compared with those that have a surplus food source and, therefore, may appear younger than they actually are [22].

Volatile organic compounds and cuticular hydrocarbons are being examined by gas chromatography and mass spectrometry (GC/MS) to age immature Calliphoridae and are showing some considerable promise [27,28,29,30,31,32,33,34]. Shortcomings do, however, arise with aging by changes in cuticular hydrocarbons and volatile organic compounds recovered from the headspace. These methods are destructive to the sample of insects and are not rapid.

Gene expression has been introduced as a viable means to age blow fly eggs, larvae, and pupae [18,35]. The gene transcript levels are examined and can be used as demarcations within stages to offer a more precise timeline. The analyzed sample is destroyed in a lengthy process and requires a trained molecular biologist and extensive equipment to carry out the gene sequencing.

Within the intra-puparial period, there are well noted morphological changes that can be used to further demarcate the stage and provide more precision when estimating insect age during metamorphosis [36,37,38]. These methods are very valuable, but time consuming and destructive to the insect sample. 3D micro-computed tomography (CT) has been used to assist in viewing the changes to the developing pupae and pharate adult during metamorphosis [39]. However, CT technology is costly and although not normally invasive except for the X-ray exposure to the sample, in this research the sample preparation was damaging.

Caveats such as larval aggregate formation increasing development temperature, inaccurate lower temperature thresholds applied to calculate the ADD/Hs, geographical developmental differences, and fluctuating temperatures changing rate of development already exist in estimating the age of immature Calliphoridae [40,41,42]. Hence, if further limitations in the methods used to estimate age can be reduced then compounded errors can be avoided. Remote sensing is a promising science with few shortcomings and is being utilized in many sciences. Remote sensing is well known for being neither invasive nor damaging and can considerably improve precision of most techniques.

Very recently, hyperspectral remote sensing has shown beneficial impact in forensic entomology by rapidly discriminating blow fly species at the larval stage [43]. Also, pushbroom hyperspectral imaging to age *Calliphora dubia* Macquart and *Chrysomya rufifacies* Macquart puparia measured dorsally and ventrally has now been completed to refine the puparial stage based on development of adult morphological characteristics [20].

Since much of the immature stages of necrophagous blow flies is spent moving on the food substrate, food contaminants may cover the surface which can pose an issue with measuring reflectance because reflectance measurements may not be of the developing larvae but instead of the decomposing tissue.

The objectives of this research were to examine hyperspectral point source measurements of developing *Lucilia sericata* (Meigen) at wavelengths that extend from 350 to 2500 nm, to identify the optimal location on the insect for applying hyperspectral measurements, and to compare measurements from washed specimens to the same unwashed specimens.

## 2. Materials and Methods

### 2.1. Insect Rearing

*Lucilia sericata* colonies were established with blow flies provided by the Simon Fraser University Department of Biological Sciences Insectary. These colonies originated from wild caught flies collected from Burnaby, Langley, and Vancouver, British Columbia and were used within a year of trapping. The blow flies were trapped using dimethyl trisulfide lures in combination with dead rats. The *L. sericata* species were confirmed using Whitworth’s keys [44] and were separated into two colonies. These colonies were maintained at room temperature (23–25 °C) in the Centre for Forensic Research, Forensic Entomology Laboratory at Simon Fraser University. These adult colonies were maintained in 75 cm^3^ cages and raised on milk powder, sugar cubes, and water ad libitum. Beef liver was added to the cages frequently to encourage reproductive development and to maintain the colony.

### 2.2. Experimental Protocol

Beef liver was used as the oviposition medium and was placed inside black film canisters positioned on their sides in each colony at approximately 1400 h [45]. To ensure that enough eggs were collected for the experiment, eggs were collected two hours after placing the canisters into the cages. The eggs from the two colonies were divided amongst four “treatments” so that each treatment received an estimated 240 combined number of eggs from both colonies. Each treatment consisted of a one gallon/four liter wide mouthed glass jar of moistened sawdust (~5 cm deep) and on top of that was placed ~250 g of freshly thawed beef liver as the larval rearing medium, positioned on a folded industrial paper towel to soak up any excess fluids. The jars were secured with two pieces of paper towel and fastened by elastic bands to prevent escape.

The treatments were maintained in a Conviron^®^ E/7 (Controlled Environments Ltd., Winnipeg, MB, Canada) environmental chamber set for 75% relative humidity and a photoperiod of 14:10 (L:D) hours. The chamber maintained a constant mean temperature of 23.9 °C and the jars were rotated daily within the chamber to account for any temperature differences. Temperature was recorded by Smartbutton^®^ data loggers (ACR Systems Inc., Surrey, BC, Canada) and checked daily using Fisherbrand™ thermometers (Thermo Fisher Scientific, Ottawa, ON, Canada).

Once the insects reached second stadium, each treatment was removed once daily, beginning at approximately noon, from the chamber for measuring with an ASD (Analytical Spectral Devices™, Boulder, CO, USA) LabSpec 4 Spectrometer. The stage of development was noted daily based on the number of spiracular slits and crop size [46], and spectral measurements of 10 insects from each of the four treatments were completed daily until adult emergence. Those same larvae/pupae were measured again after washing with deionized water and filter paper and then blotting dry with filter paper in order to ensure that the measurements taken were of the insect and not from the feeding media on the insect surface. In order to keep the insects alive and not to affect reflectance measures, this is modified slightly from the suggested washing techniques of deionized water (tap water)/filter paper/freezing [47,48], deionized water/0.9% NaCl solution [47,49], and distilled water/methanol [49,50]. Excess water was removed with dry filter paper from the insect surface because water exhibits specular reflections and could be misleading to the spectral signature [51].

**Figure 1:**
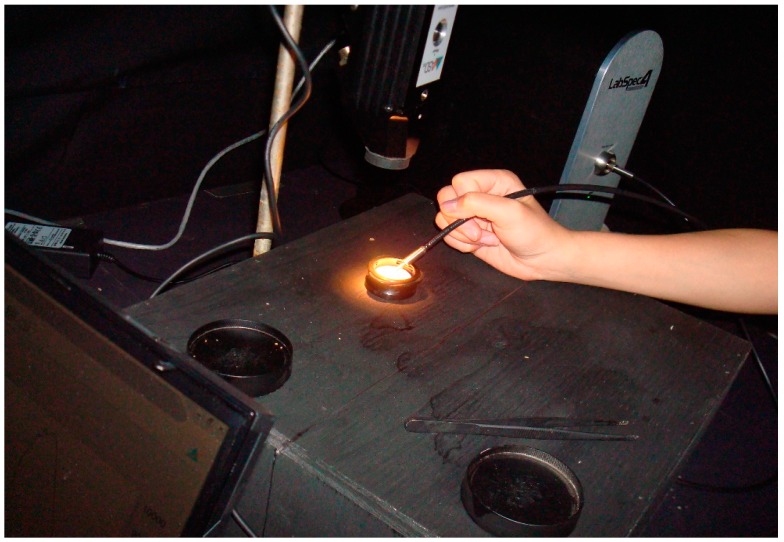
Spectrometer set up in the blackened laboratory and calibration using a Spectralon panel.

**Figure 2:**
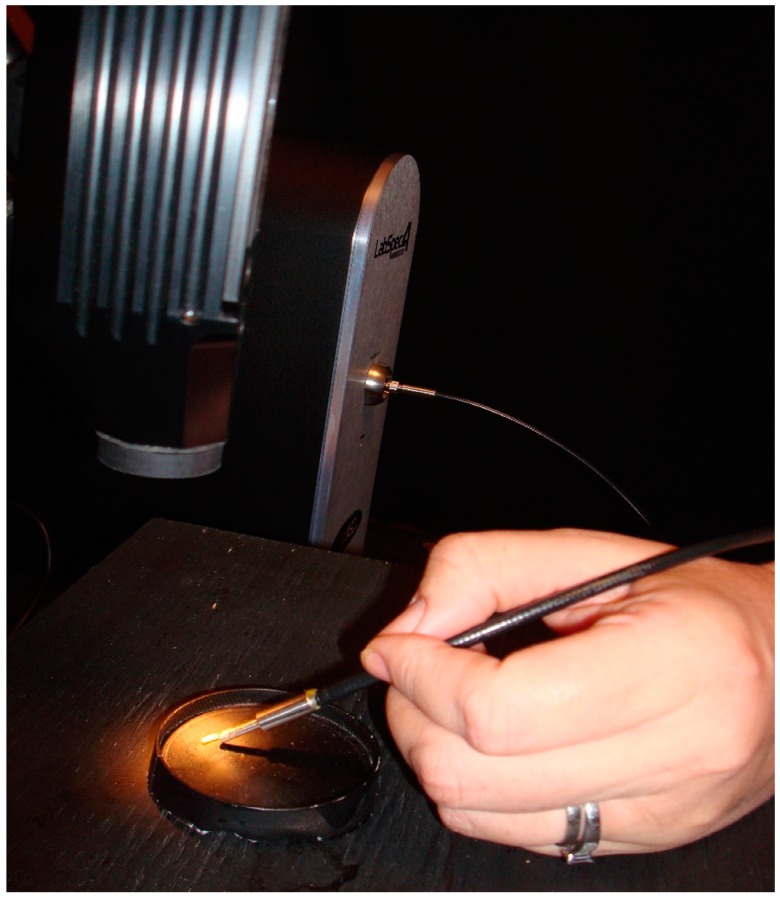
Demonstration of measuring reflectance from an insect with the Analytical Spectral Devices (ASD) Labspec 4 Spectrometer.

All reflectance measurements were performed in a blackened laboratory. The light source used was an ASD lower intensity Welch Allyn plant probe bulb (lux = 968 at 0.9 mm and 4.25 v but used at a distance of 8.5 cm and a reduced voltage of 3.825 v) in a contact probe. Negligible extraneous light did enter the room from the computer screen which was turned away from the measuring area and from under the door; this was consistent in all measurements. The walls were draped with black fabric and the insect platform on which the measurements were carried out was painted flat black (General paint fast drying spray enamel), as was all the equipment including petri dishes and forceps (Figure 1 and Figure 2). This was done to ensure that measurements were strictly of the insect and not of any reflecting surrounding surfaces and was verified with the spectrometer. For each measurement taken, the reflectance was recorded from wavelengths ranging from 350 to 2500 nm. All measurements were equidistant (8.5 cm) from the 90 degree light source and taken at approximately a 30 degree angle from the insect platform. There was more saturated light within the central 3 cm diameter of the field of light and so that was where the measurements were consistently taken. The light source was originally tested at a height of 5.5 cm above the insects, but the larvae were dying under the light following washing, so the height was extended to 8.5 cm to avoid this.

Three point measurements were taken from each unwashed and then washed insect, from the anterior, middle, and posterior regions of the insect. Before insect measurements were taken, and following every five to seven measurements, calibration with a pure diffuse reflectance standard was completed to provide a baseline. In this case, a Spectralon™ panel was used to calibrate the spectrometer (Figure 1). During the process of optimization, a black reference was also completed. Optimization of the spectrometer is necessary to accommodate the integration time and counterbalance the short-wave infrared.

These data were stored as files specific to the RS3™ software (Analytical Spectral Devices, Boulder, CO, USA) and so Viewspec pro™ (Analytical Spectral Devices, Boulder, CO, USA) was used to convert them to usable text files. These text files were then organized into a Microsoft Office™ Excel (Microsoft, Redmond, WA, USA) template to organize them by day and body region before transferring them into Matlab (Mathworks™, Natick, MA, USA) for statistical analysis along with fdaM (functional data analysis Matlab) tools (http://www.psych.mcgill.ca/misc/fda/downloads/FDAfuns/).

### 2.3. Pre-Processing

The original data had a particular artifact. Specifically, reflectance curves tended to be very smooth with the exception of a few abrupt vertical shifts. These vertical shifts were not unique to each individual insect, but were unique to a given day of measuring and occurred at the same wavelength(s) for each individual. For a set of reflectance curves X(w), over wavelength, w, the first differences: X(w + 1) − X(w), were scanned and the wavelength with the largest maximum absolute difference over all individuals was flagged as a jump point or vertical shift. At the jump point, all individuals were processed based on the amount of their individual shift such that:

X(w + 1: end) = X(w + 1: end) − (X(w+1) − X(w))
(1)

In general, two such jump points were present in the data from each measurement. These jump points were not in any particular direction and consisted of different amplitudes for different individuals. It is probable that there may have been a crimp in the PVC (a synthetic polymer of poly vinyl chloride) fiber optic cable that went unnoticed while measuring, which caused the jump point.

The reflectance levels were internally calibrated for each reflectance curve for each measured insect. In order to calibrate the baseline measurement, the reflectance curves were first smoothed and then the smooth curves were vertically shifted so that they take an average value of 0 reflectance over the wavelength interval (400, 550 nm). The curves were then rescaled by setting the maximum value of reflectance to 1.

### 2.4. Functional Linear Model to Estimate Day of Development

#### 2.4.1. Smoothing Individual Reflectance Measurements

Individual reflectance functions were smoothed using 6th order B-Spline basis functions, Φ(w), with 100 evenly spaced knots [52]. The coefficients, C = [C_1_,…, C_J_] were acquired in order to approximate the reflectance functions X(w) for the basis expansion: X(w) ≈ X_smooth_(w) = $\sum_{j=1}^{J}$ Cj Φj(w) [52]. A curvature penalty was generated to avoid bias from over-fitting the functions [52]:

(2) $${\mathrm{PEN}}_{x}=\int_{350}^{2500}{\left(\frac{d^{2}}{dw^{2}}X_{smooth}\left(w\right)\right)}^{2}dw$$

For all days at each body region, (anterior, mid-section, and posterior) a tuning parameter λ was applied [52]:

(3) $$\begin{array}{c} \mathrm{argmin}\\ C \end{array}[\sum_{350}^{2500}{\left(X_{smooth}\left(w\right)-X\left(w\right)\right)}^{2}+\lambda \int_{350}^{2500}{\left(\frac{d^{3}}{dw^{3}}X_{smooth}\left(w\right)\right)}^{2}dw],$$

where $\begin{array}{c} \mathrm{argmin}\\ C \end{array}$ is the argument of the minimum coefficient. Generalized Cross Validation was used to tune *λ* uniquely for each body region to avoid overfitting while reducing the noise found within the original spectra [52].

#### 2.4.2. Functional Linear Model with Scalar Response

The multivariate functional linear model:

(4) $$Y_{i}=\int_{350}^{2500}X_{i,smooth}\left(w\right)\beta \left(w\right)dw$$

estimates the functional covariate *β*(*w*) which best predicts the age of insect (Y*_i_*) for each body region. In this case, a third order derivative penalty, PEN*_β_* was used to penalize the function [52],

(5) $${\mathrm{PEN}}_{\beta }=\int_{350}^{2500}{\left(\frac{d^{2}}{dw^{2}}\beta \left(w\right)\right)}^{2}dw,$$

with its own smoothing parameter, *λ_β_* to prevent overfitting the regression model for the data. Leave-one-out cross validation was used to estimate λ*_β_* [52]. The Sum of Squares Cross Validation for the fixed value of *λ_β_*:

(6) $$\underset{i}{\sum }{\left({\hat{Y}}_{i}-Y_{i}\right)}^{2}=\underset{i}{\sum }{\left(\int_{350}^{2500}X_{i,smooth}\left(w\right)\beta_{-i}\left(w\right)dw-Y_{i}\right)}^{2}$$

is minimized to choose the optimal *λ_β_* that avoids overfitting the data [52]. The resulting estimate of *β*(*w*) and *λ_β_* can then be used to predict new insect ages, Y, based on new wavelength reflectance X(w). The bootstrap 95% confidence intervals with upper and lower limits were applied to each functional regression using Matlab R2015b.

#### 2.4.3. Model Fitting to Compare Washed to Unwashed Insects

To compare the spectral measurements of the unwashed insects to the spectral measurements of the washed insects an extension of the previous model was used:

(7) $$Y_{i}=\int_{350}^{2500}X_{i,smooth}\left(w\right)[\beta {\left(w\right)}_{was}+I_{unw}\beta {\left(w\right)}_{unw}]dw.$$

This extension allows the examination of the functional regression coefficients of the washed insects *β*(*w*)*_was_*,

(8) $$Y_{i}=\int_{350}^{2500}X_{i,smooth}\left(w\right)\;\beta {\left(w\right)}_{was}dw,$$

and the impact of coefficients of the unwashed insects, *β*(*w*)*_unw_*,

(9) $$Y_{i}=\int_{350}^{2500}X_{i,smooth}\left(w\right)\;[\beta {\left(w\right)}_{was}+\beta {\left(w\right)}_{unw}]dw.$$

An examination of the coefficients was used to test the null hypothesis *β*(*w*)*_unw_* = 0 to see if there is an advantage to washing the insects before collecting spectral measurements.

## 3. Results

### 3.1. Spectral Analysis

At an average temperature of 23.9 °C, *L. sericata* spent one day as second instar, one day as third instar, and then six days as a post-feeding third instar larvae before entering the intra-puparial period. The intra-puparial period lasted five days and on day 6 the adults began emerging. Spectral measurements were collected for *L. sericata* from each of these days of development for each of the anterior end, midsection, and posterior end of the insects.

At each of the anterior end, midsection, and posterior end, the daily spectral signatures were examined for the lengthy post-feeding stage, the intra-puparial period and then, as there was only a single day for each of the second and feeding third instar periods, a comparison was made between those two stages from the midsection measurements.

**Figure 3:**
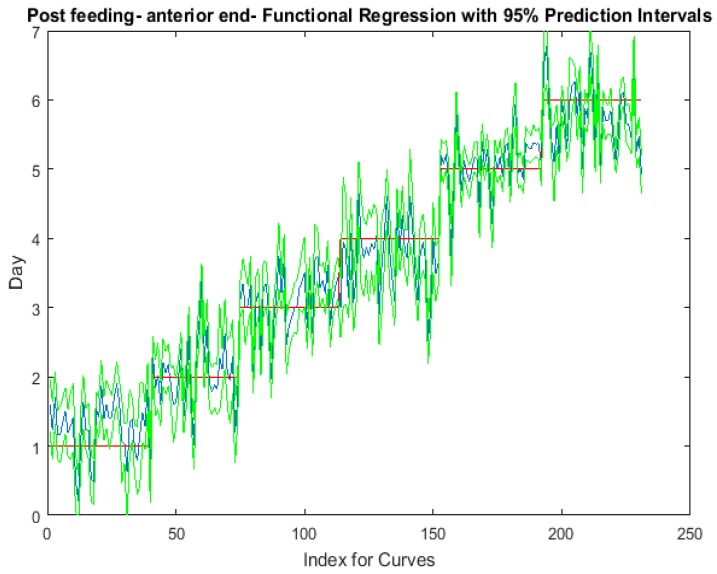
The actual (red) versus predicted days (blue) for *Lucilia sericata* post-feeding larvae (raised at a mean temperature of 23.9 °C) spectral measurements from 350–2500 nm of the anterior end. The pointwise 95% prediction interval upper and lower limits appear in green.

**Figure 4:**
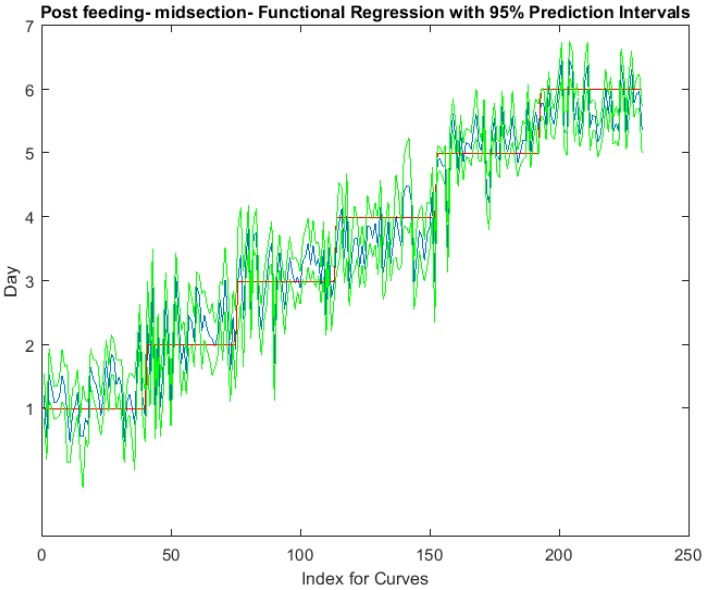
The actual (red) versus predicted days (blue) for *Lucilia sericata* post-feeding larvae (raised at a mean temperature of 23.9 °C) spectral measurements from 350 to 2500 nm of the midsection. The pointwise 95% prediction interval upper and lower limits appear in green.

**Figure 5:**
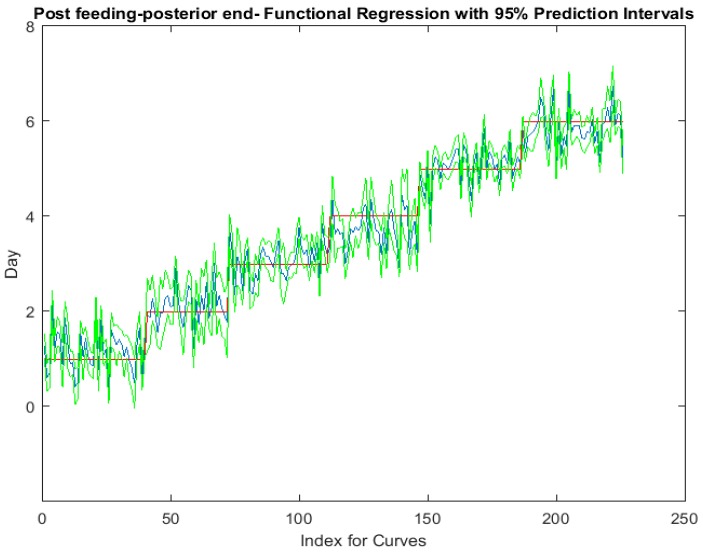
The actual (red) versus predicted days (blue) for *Lucilia sericata* post-feeding larvae (raised at a mean temperature of 23.9 °C) spectral measurements from 350 to 2500 nm of the posterior end. The pointwise 95% prediction interval upper and lower limits appear in green.

The functional regressions for each model based on the post-feeding stage of the washed larvae indicate that each of the models from all three measured regions are a good predictor of day within the post-feeding stage (Figure 3, Figure 4 and Figure 5). The spectral measurements from the posterior region of the larvae provide a superior prediction, compared with the anterior and midsection measurements.

**Figure 6:**
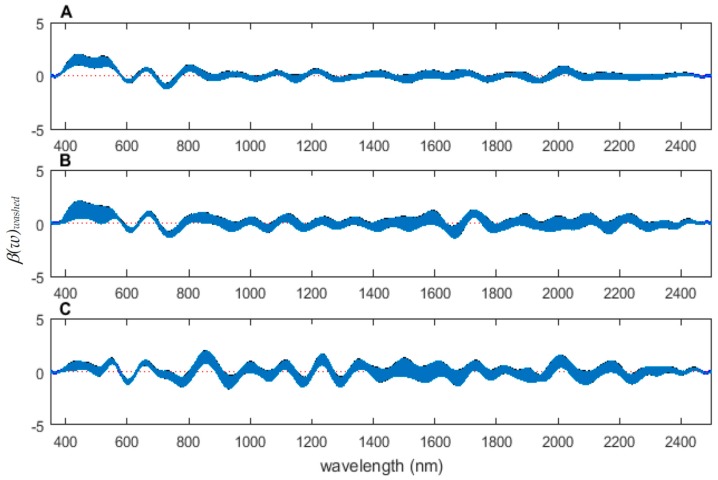
The contributing *β*(w)_washed_ coefficients of the spectral measurements for each of the measured regions ((**A**) anterior end, (**B**) midsection, and (**C**) posterior end) of *Lucilia sericata* post-feeding larvae raised at a mean temperature of 23.9 °C.

The coefficient functions are presented in Figure 6 and indicate that the model is strong for predicting age because the *β*(*w*) coefficient did not overlap with zero over all wavelength regions. This is evident because the red zero line is visible, which indicates that there is a slope. Not only do the coefficient functions indicate that the slope of the linear function is not zero, but they also indicate the contributing wavelengths of the electromagnetic spectrum to the model for each measured body region.

**Figure 7:**
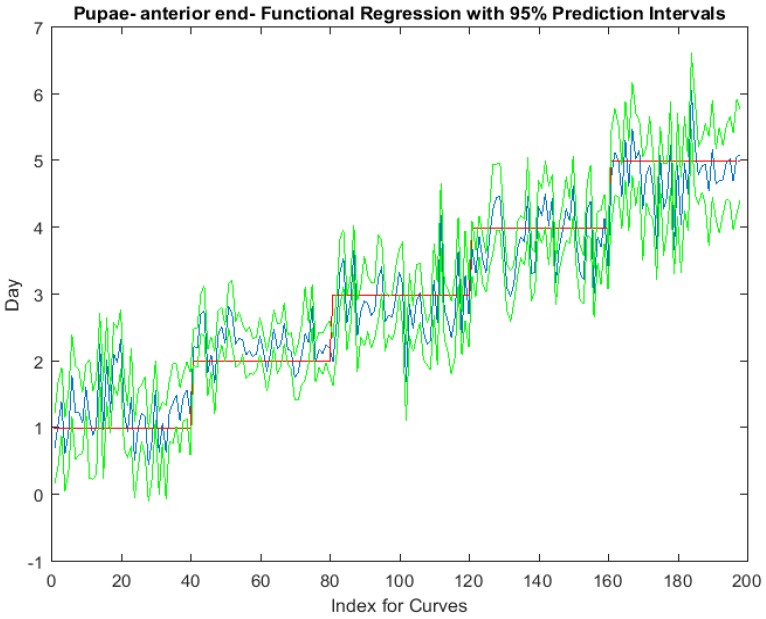
The functional regression with 95% upper and lower limit pointwise prediction intervals (green) for spectral measurements from 350 to 2500 nm of the anterior end of *Lucilia sericata* pupae raised at a mean temperature of 23.9 °C. The predicted days appear in blue and the red line is the actual day.

**Figure 8:**
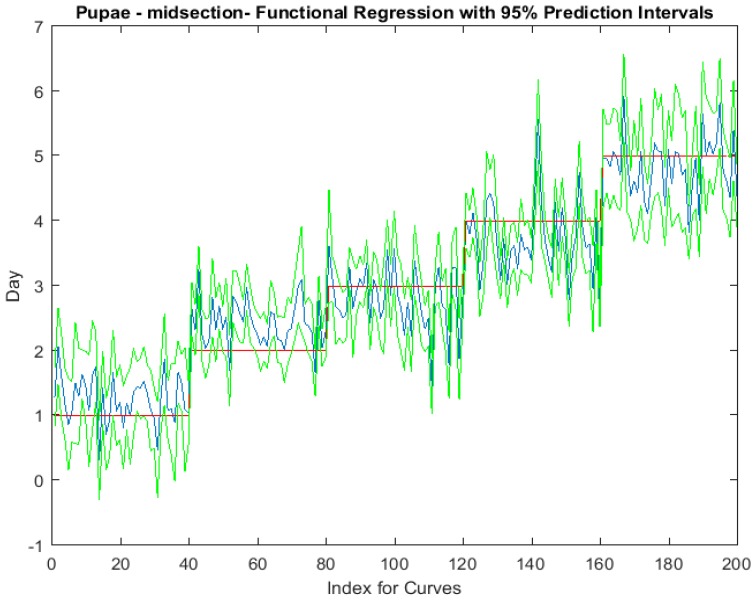
The functional regression with 95% upper and lower limit pointwise prediction intervals (green) for spectral measurements from 350 to 2500 nm of the midsection of *Lucilia sericata* pupae raised at a mean temperature of 23.9 °C. The predicted days appear in blue and the red line is the actual day.

**Figure 9:**
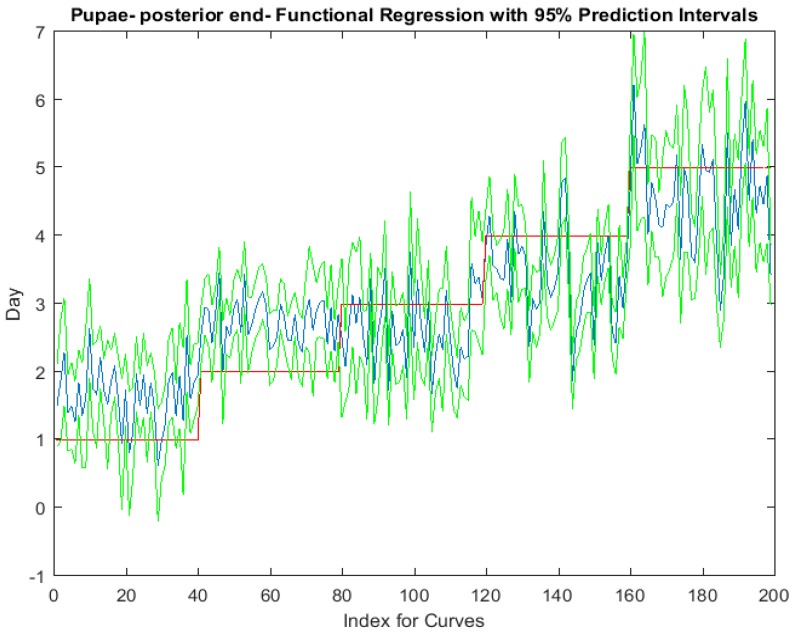
The functional regression with 95% upper and lower limit pointwise prediction intervals (green) for spectral measurements from 350 to 2500 nm of the posterior end of *Lucilia sericata* pupae raised at a mean temperature of 23.9 °C. The predicted days appear in blue and the red line is the actual day.

Examining the intra-puparial functional regressions from spectral signatures that range from 350 to 2500 nm does show some distinction between the days (Figure 7, Figure 8 and Figure 9). The anterior region of the pupa offers the best prediction of day in the intra-puparial period. The first three days of the intra-puparial period is not clearly predicted from the spectral measures of the midsection and posterior regions.

**Figure 10:**
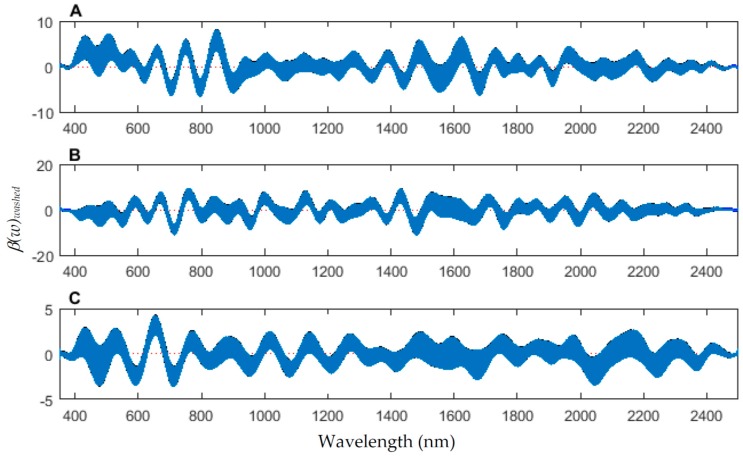
The contributing coefficients, *β*(*w*)_washed_ of the spectral measurements for each of the measured regions ((**A**) anterior end, (**B**) midsection; and (**C**) posterior end) of *Lucilia sericata* pupae raised at a mean temperature of 23.9 °C.

The coefficient functions indicate that there is a contributing *β*(*w*) coefficient to the function for each measured body region, and also indicate that there are different contributing wavelengths to the spectral signatures for each of the body regions (Figure 10).

**Figure 11:**
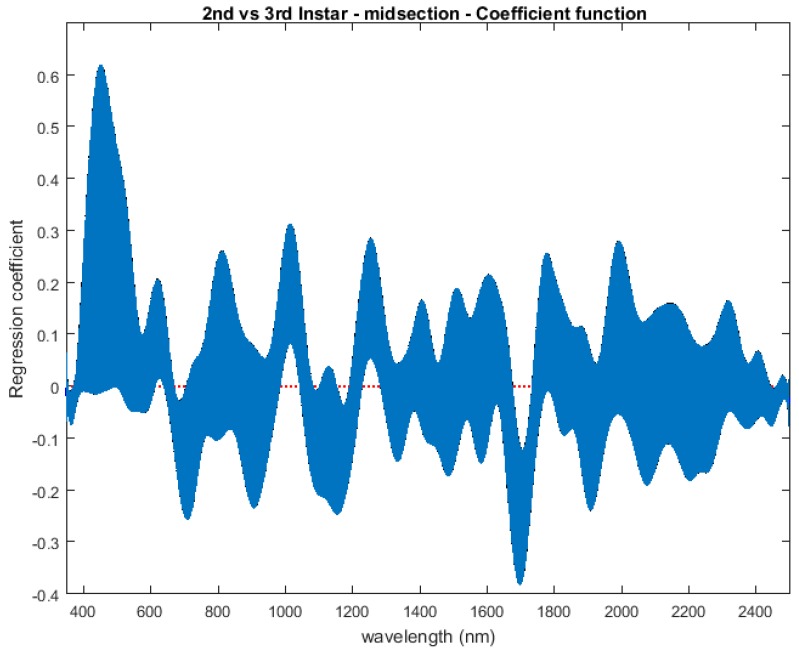
The coefficients with their 95% confidence bands contributing to the model that compares spectral measurements from the one day of second instar with the one day of third instar *Lucilia sericata* raised at a mean temperature of 23.9 °C. It also indicates the wavelengths that contribute to the daily prediction.

**Figure 12:**
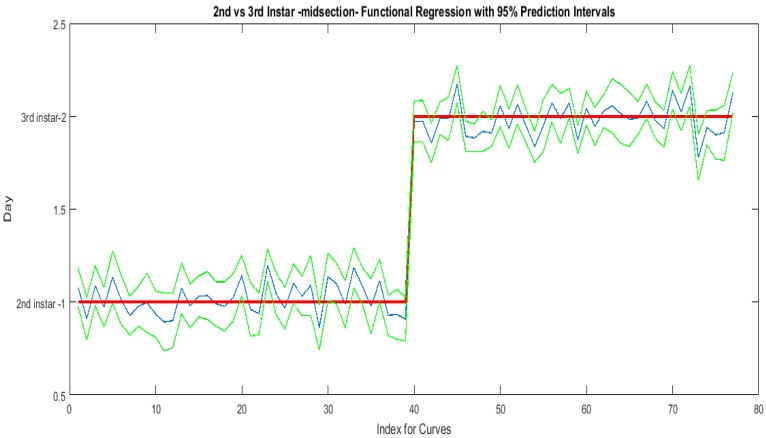
The actual (red) versus predicted days (blue) for *Lucilia sericata* second and third instar larvae (raised at a mean temperature of 23.9 °C) spectral measurements of the midsection. The pointwise 95% prediction interval upper and lower limits appear in green.

The contributing wavelengths and coefficients for distinguishing second instar from third instar are presented in Figure 11. The functional regression based on midsection spectral measurements easily predicts day one (second instar) from day two (third instar) (Figure 12). The predicted days hover over the actual day and fall within the 95% prediction upper and lower limits.

**Figure 13:**
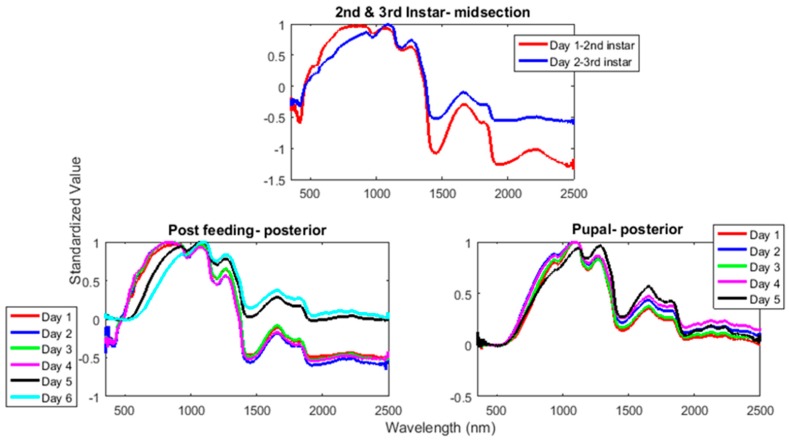
The mean preprocessed smoothed spectral measurement plots for each day of each stage of *Lucilia sericata* raised at a mean temperature of 23.9 °C.

The mean preprocessed smoothed plots for each day within the stages from second instar until adult emergence from *L. sericata* raised at a mean temperature of 23.9 °C are presented in Figure 13 and show the daily differences in spectral measurements. Troughs that are observed at ~550 and 950 nm in the second instar both disappear before intra-puparial development is reached.

### 3.2. Washed Versus Unwashed

**Figure 14:**
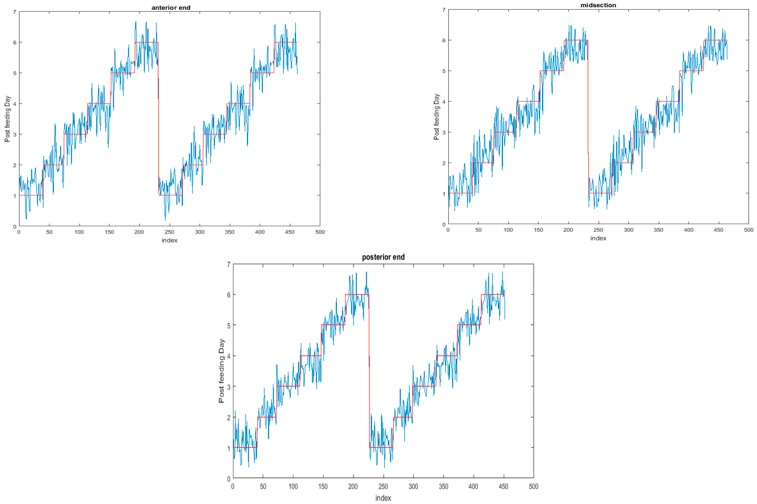
The functional regression models of unwashed *Lucilia sericata* raised at a mean temperature of 23.9 °C and the same washed post-feeding larval anterior end/midsection/posterior end spectral measurements. The red line indicates the actual day and the blue indicates the predicted day. The washed predictions appear on the left of each plot and the unwashed on the right.

**Figure 15:**
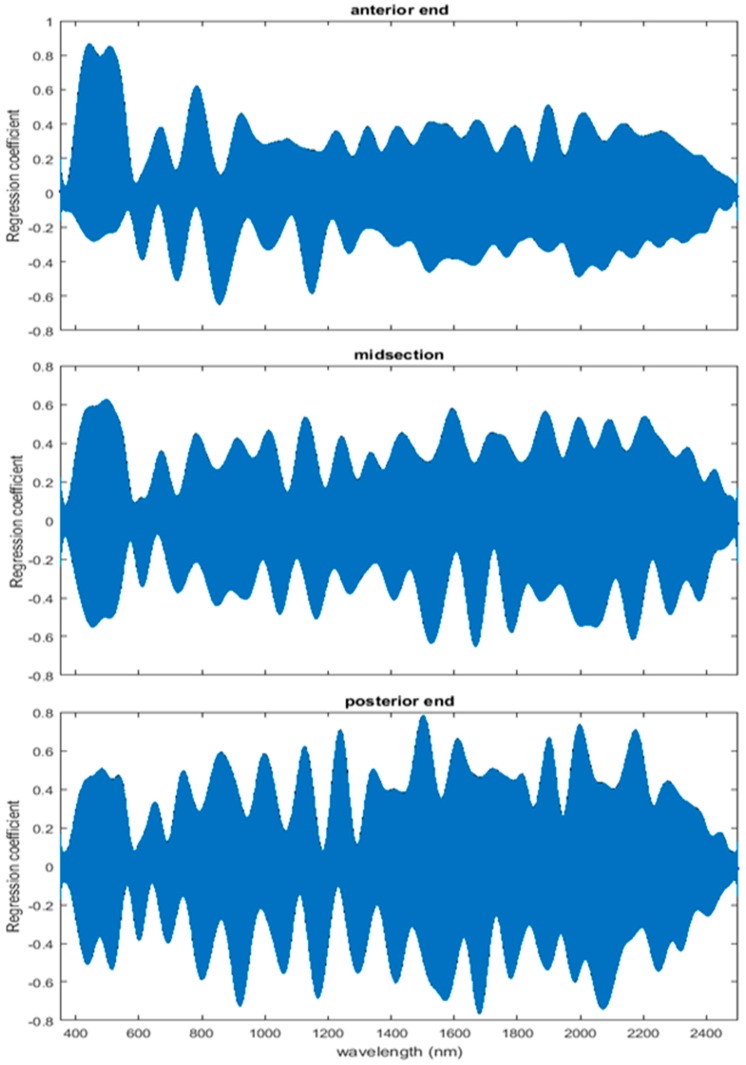
The 95% confidence interval of the coefficients for post-feeding *Lucilia sericata* raised at a mean temperature of 23.9 °C in a model predicting the effect of lack of washing before taking spectral measurements (350–2500 nm) from the anterior end/midsection/posterior end of the post-feeding larvae.

To examine the necessity of washing the larvae for the spectral measurements, an analysis of the impact of the unwashed larvae spectral measurements on the washed larvae measurements was completed for each of the larval stages. Applying the unwashed post-feeding larvae spectral measurements to each of the three models has no significant effect (*p* < 0.05) on the models. This was evident when examining the functional regressions based on all measured regions (Figure 14). At no wavelength does the addition of unwashed measurements change the prediction. The slopes of the coefficients are almost zero and do not contribute to the model (Figure 15). Thus, this implies that there is no significant difference when including the unwashed data when it comes to predicting the day within the post-feeding stage.

**Figure 16:**
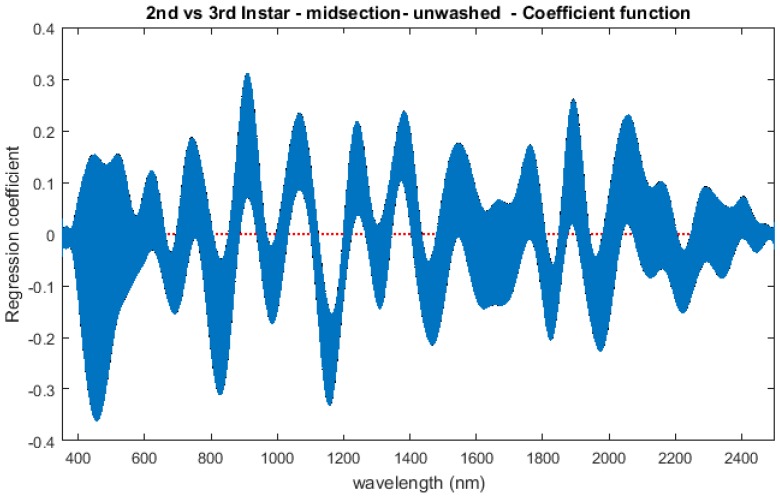
The second and third instar *Lucilia sericata* (raised at a mean temperature of 23.9 °C) function of the unwashed coefficients with 95% confidence bands for the midsection spectral measurements (350–2500 nm). The contributing wavelengths are indicated by the dotted red zero line.

**Figure 17:**
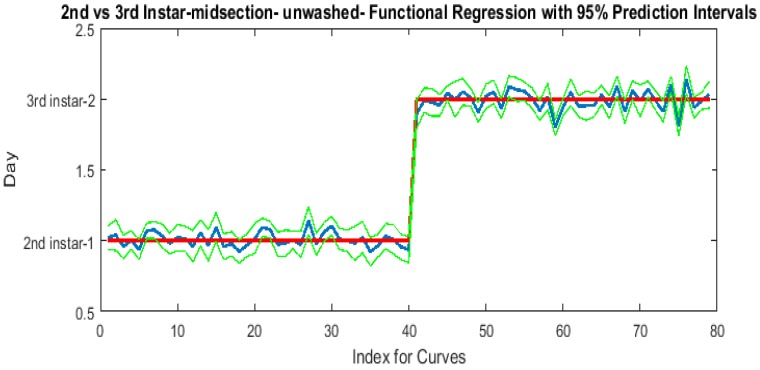
The functional regression for predicting second and third instar *Lucilia sericata* (raised at a mean temperature of 23.9 °C) from unwashed larvae based on spectral measurements. The red line is the actual day, the blue line is the prediction, and the green lines are the upper and lower 95% pointwise prediction intervals.

An examination of whether washing is necessary in the feeding larval stages was also completed, as this would presumably affect the spectral measurements since the larvae are moving through the food source. The contributing coefficients of the washed specimens in Figure 11 differ from those of the unwashed specimens in Figure 16. There are more contributing wavelengths in the unwashed specimens and at those wavelengths the *β*(*w*) coefficients are significant (*p* < 0.05). However, the functional regression of the unwashed specimens (Figure 17) predicts the different days just as consistently within 95% confidence intervals as the functional regression of the washed specimens (Figure 12). Including unwashed specimens in the analysis does affect the analysis, but the findings are the same.

## 4. Discussion

Differences were found in the daily spectral signatures of the post-feeding stage of *L. sericata*. The posterior spectral measurements surpassed the anterior and midsection measurements for prediction of day in the post-feeding stage. There were different contributing wavelengths and coefficients for the different regions of the insect (Figure 6). The posterior end had the most contributing wavelengths to the model. In fact, while measuring, it was noted that daily changes were visible in the posterior spectral measurements before they were visible in the midsection and anterior measurements. The position of oenocytes probably indirectly explains most of these findings. They are regularly found in the insect abdominal integument proximal to the spiracles [53]. Oenocytes produce cuticular hydrocarbons, which are then transported to the remainder of the cuticle and fat body by lipophorin in the hemolymph [54]. Cuticular hydrocarbons waterproof the insect and contribute to the near infrared (NIR) and shortwave infrared (SWIR) spectral measurements of the insect surface [55]. They also have been found to vary in necrophagous insects within larval stages [27,29,30,31]. Since the location of the oenocytes are most probably in the abdomen, cuticular hydrocarbons are probably identified earlier and of a stronger concentration than they are at the further away body regions of the anterior end and midsection. Besides hydrocarbons, color or opaqueness, surface texture, tracheal changes near to the light penetrable surface, mouthpart and spiracular changes all may contribute in some form to the spectral measurements.

In the intra-puparial period, the best daily prediction was provided by the anterior measurements. This was probably due to the changes that occur to the brain and blow fly ommatidia appearance, which are much more immediate in metamorphosis than those changes that occur in the remaining insect body, which remains obscured longer by the fat body [37]. It was very probable that light was able to perforate the puparium by the chitinous pore canals that extend along the endocuticle from which the puparium has formed. The outer portion of the very thick endocuticle is formed by secretion during the larval stages and it is the outer endocuticle which is perforated by the pore canals. This outer endocuticle with pore canals is then predetermined to form the puparium in the intra-puparial period [56]. The pore canals form a vertical striation in the puparium [57] and it potentially may be these striations that allow light to penetrate the puparium to detect these changes. Unlike this research, previous researchers examined spectral imaging of the dorsal and ventral surfaces of blow fly puparia ranging from 389 to 892 nm in relation to morphological changes and found that both surfaces were well within 80% accurate [20].

In a few pointwise 95% confidence interval daily predictions the actual day did not fully fall within the upper and lower limits, but was very close. These outlier measurements may be explained by slight development differences within replicates. These slight variations may be explained by time differences outside of the environmental chamber or temperature variations due to placement of the replicate within the chamber. To avoid temperature variations within the chamber, replicates were rotated, but replicates may still have been exposed to slight differences.

Simpler methods already exist to distinguish between second and third instar larvae, such as examining the number of spiracular slits. Examining the earlier stadia with spectral measurements, although accurate, is not necessary at higher temperatures, but may be useful at lower temperatures when the second and third instar stadia extend over multiple days. Figure 13 presents the daily mean preprocessed spectral signatures for each day of development at a mean of 23.9 °C. The figure shows the loss of a trough at 550 nm in the second and third instar measurements and the loss of a second trough during the intra-puparial period. The first trough is completely gone by the intra-puparial period (550 nm), and the second is almost missing by the last day of intra-puparial development before the adult emerges (950 nm). The disappearance of both troughs is probably due to water loss or absorption from the cuticle. In the intra-puparial period, a substantial amount of water loss occurs in the cuticle, in order to reinforce the puparium as it forms [58].

Unusual vertical jump points were found in some of the raw collected spectral data in a few of the collected days of data. They were not found consistently and were not a component of a regular measurement, as there are smooth transitions from wavelength to wavelength in reflectance [59]. Hence the only explanation can be human or machine error. There may have been a crimp within the fiber optic cable that went unnoticed. Even with this vertical jump that appeared in one day of measuring, correction was easily managed.

A noteworthy detail was that the spectral signature from the last day of one stage was basically the same as the spectral signature from the first day of the next stage. By examining the spiracular slits and combining this with the knowledge of what the insect’s spectral reflectance signature is in the following stage, conclusions can be made that the insect is at the end of a stage rather than the beginning. Rather than using the minimum time it takes to reach a stage it would be more appropriate to examine the time spent in a stage at that developing temperature.

Washing the newly molted third instar larvae with deionized water and filter paper, blotting dry with clean dry filter paper, and then placing under the light source before taking the spectral measurements was distressing to the larvae at this stage only. At first it was thought that it was the heat of the light source, so the light source was moved from 5.5 cm height to 8.5 cm, but this did not correct this issue. It was later believed that the washing following the third instar molt was in fact the issue. By being very gentle with the third instar larvae, it was possible to keep the larvae alive for measurement.

Washing the post-feeding larvae made no difference to the contributing beta (*β*) coefficients. There was no advantage to washing the insects in the wandering post-feeding stage. However, the *β* coefficients of the unwashed second and third instar larvae did differ from the washed. Measurements of second and third instar larvae are likely to be the most affected due to the larvae moving through the food source and collecting food contaminants on their cuticle. Nevertheless, the predictions of the washed and unwashed were both excellent and within 95% prediction interval upper and lower limits, and so suggest that washing the specimens does not affect prediction of day of development. Washing did, however, affect the recent feeding third instar larvae. High mortality rates were experienced when washing the newly molted third instar *L. sericata* larvae. It is probable that because they were newly molted, the third instar larvae were more susceptible to washing.

## 5. Conclusions

Hyperspectral measurements of the surface of *L. sericata* larvae can be used to distinguish day within the lengthy post-feeding stage quite readily and a satisfactory estimate from the intra-puparial period can also be estimated using:

(10) $$Y=\int_{350}^{2500}X\left(w\right)*\beta \left(w\right)dw,$$

where *Y* is the predicted day in the stage, *X* is the spectral measurement from 350 to 2500 nm, and *β*(*w*) is the contributing coefficients of the function.

It is recommended to use posterior measurements for post-feeding larvae and anterior measurements for pupae. Washing the post-feeding larvae before taking spectral measurements does not seem to offer any benefit, which is not surprising since the larvae have moved away from the food source. Washing does make a difference to the coefficients for the feeding second and third instar larval spectral measurements, but the predictions are not changed by the lack of washing and closely fit the upper and lower limits of the 95% confidence intervals. Nevertheless, it is recommended that surface contaminants be gently washed from the insect cuticle, if possible, so that the spectral daily signatures can best be individualized.

## References

[1] Nansen C., Ribeiro L.P., Dadour I., Roberts J.D. (2015). Detection of temporal changes in insect body reflectance in response to killing agents. PLoS ONE.

[2] Wilson B.C., Jacques S.L. (1990). Optical reflectance and transmittance of tissues: Principles and applications. IEEE J. Quantum Electron..

[3] Carroll M.W., Glaser J.A., Hellmich R.L., Hunt T.E., Sappington T.W., Calvin D., Copenhaver K., Fridgen J. (2008). Use of spectral vegetation indices derived from airborne hyperspectral imagery for detection of european corn borer infestation in Rowa corn plots. J. Econ. Entomol..

[4] Hart W.G., Ingle S.J., Davis M.R., Mangum C., Higgins A., Boling J.C. Some uses of infrared aerial color photography in entomology. Proceedings of the Third Biennial Workshop on Aerial Color in the Plant Sciences.

[5] Lawrence R., Labus M. (2003). Early detection of Douglas-fir beetle infestation with subcanopy resolution hyperspectral imagery. West. J. Appl. For..

[6] Mirik M., Ansley R.J., Steddom K., Rush C.M., Michels G.J., Workneh F., Cui S., Elliott N.C. (2014). High spectral and spatial resolution hyperspectral imagery for quantifying russian wheat aphid infestation in wheat using the constrained energy minimization classifier. J. Appl. Remote Sens..

[7] Mirik M., Michels G.J., Kassymzhanova-Mirik S., Elliot N.C., Bowling R. (2006). Hyperspectral spectrometry as a means to differentiate uninfested and infested winter wheat by greenbug (Hemiptera: Aphididae). J. Econ. Entomol..

[8] Mirik M., Michels G.J., Kassymzhanova-Mirik S., Elliot N.C., Catana V., Jones D.B., Bowling R. (2006). Using digital image analysis and spectral reflectance data to quantify damage by greenbug (Hemiptera: Aphididae) in winter wheat. Comput. Electron. Agric..

[9] Moran M.S., Inoue Y., Barnes E.M. (1997). Opportunities and limitations for image-based remote sensing in precision crop management. Remote Sens. Environ..

[10] Riley J.R. (1989). Remote sensing in entomology. Annu. Rev. Entomol..

[11] Singh C.B., Jayas D.S., Paliwal J., White N.D.G. (2009). Detection of insect-damaged wheat kernels using near-infrared hyperspectral imaging. J. Stored Prod. Res..

[12] Solberg S., Eklundh L., Gjertsen A.K., Johansson T., Joyce S., Lange H., Naesset E., Olsson H., Pang Y., Solberg A. (2007). Testing remote sensing techniques for monitoring large scale insect defoliation. Remote Sens. Environ..

[13] Williams D.W., Bartels D.W., Sawyer A.J., Mastro V. Application of hyperspecral imaging to survey for emerald ash borer. Proceedings of XV U.S. Department of Agriculture Interagency Research Forum on Gypsy Moth and other invasive species Annapolis Maryland.

[14] Xing J., Guyer D., Ariana D., Lu R. (2008). Determining optimal wavebands using genetic algorithm for detection of internal insect infestation in tart cherry. Sens. Instrum. Food Qual..

[15] Beck L.R., Lobitz B.M., Wood B.L. (2000). Remote sensing and human health: New sensors and new opportunities. Emerg. Infect. Dis..

[16] Brown H.E., Diuk-Wasser M.A., Guan Y., Caskey S., Fish D. (2008). Comparison of three satellite sensors at three spatial scales to predict larval mosquito presence in connecticut wetlands. Remote Sens. Environ..

[17] Nansen C., Coelho A., Viera J.M., Parra J.R.P. (2014). Reflectance-based identification of parasitized host eggs and adult *Trichogramma* specimens. J. Exp. Biol..

[18] Tarone A.M., Foran D.R. (2011). Gene expression during blow fly development: Improving the precision of age estimates in forensic entomology. J. Forensic Sci..

[19] Catts E.P. (1992). Problems in estimating the postmortem interval in death investigations. J. Agric. Entomol..

[20] Voss S.C., Magni P., Dadour I., Nansen C. (2016). Reflectance-based determination of age and species of blowfly puparia. Int. J. Leg. Med..

[21] Tarone A.M., Foran D.R. (2008). Generalized additive models and *Lucilia sericata* growth: Assessing confidence intervals and error rates in forensic entomology. J. Forensic Sci..

[22] Anderson G.S. (2000). Minimum and maximum development rates of some forensically important Calliphoridae (Diptera). J. Forensic Sci..

[23] Reibe S., Doetinchem P.V., Madea B. (2010). A new simulation-based model for calculating post-mortem intervals using developmental data for *Lucilia sericata* (Dipt.: Calliphoridae). Parisitol. Res..

[24] Amendt J., Krettek R., Zehner R. (2004). Forensic entomology. Naturwissenschaften.

[25] Day D.M., Wallman J.F. (2006). Width as an alternative measurement to length for post-mortem interval estimations using calliphora augur (Diptera: Calliphoridae) larvae. Forensic Sci. Int..

[26] Wells J.D., Lamotte L.R., Byrd J.H., Castner J.L. (2010). Chapter 9: Estimating the Postmortem Interval. The Utility of Arthropods in Legal Investigations.

[27] Butcher J.B., Moore H.E., Day C.R., Adam C.D., Drijfhout F.P. (2013). Artificial neural network analysis of hydrocarbon profiles for the ageing of *Lucilia sericata* for post mortem interval estimation. Forensic Sci. Int..

[28] Frederickx C., Dekeirsschieter J., Brostaux Y., Wathelet J.-P., Verheggen F.J., Haubruge E. (2012). Volatile organic compounds released by blowfly larvae and pupae: New perspectives in forensic entomology. Forensic Sci. Int..

[29] Moore H.E. (2013). Analysis of Cuticular Hydrocarbons in Forensically Important Blowflies Using Mass Spectrometry and Its Application in Post Mortem Interval Estimations. Doctoral Dissertation.

[30] Moore H.E., Adam C.D., Drijfhout F.P. (2013). Potential use of hydrocarbons for aging *Lucilia sericata* blowfly larvae to establish the postmortem interval. J. Forensic Sci..

[31] Moore H.E., Adam C.D., Drijfhout F.P. (2014). Identifying 1st instar larvae for three forensically important blowfly species using “fingerprint” cuticular hydrocarbon analysis. Forensic Sci. Int..

[32] Pechal J.L., Moore H.E., Drijfhout F., Benbow M.E. (2014). Hydrocarbon profiles throughout adult Calliphoridae aging: A promising tool for forensic entomology. Forensic Sci. Int..

[33] Xu H., Ye G.-Y., Xu Y., Hu C., Zhu G.-H. (2014). Age-dependent changes in cuticular hydrocarbons of larvae in *Aldrichina grahami* (Aldrich) (Diptera: Calliphoridae). Forensic Sci. Int..

[34] Zhu G.H., Xu X.H., Yu X.J., Zhang Y., Wang J.F. (2007). Puparial case hydrocarbons of *Chrysomya megacephala* as an indicator of the postmortem interval. Forensic Sci. Int..

[35] Tarone A.M., Jennings K.C., Foran D.R. (2007). Aging blow fly eggs using gene expression: A feasibility study. J. Forensic Sci..

[36] Brown K., Thorne A., Harvey M. (2015). *Calliphora vicina* (Diptera: Calliphoridae) pupae: A timeline of external morphological development and a new age and PMI estimation tool. Int. J. Leg. Med..

[37] Davies K., Harvey M.L. (2013). Internal morphological analysis for age estimation of blow fly pupae (Diptera: Calliphoridae) in postmortem interval estimation. J. Forensic Sci..

[38] Defilippo F., Bonilauri P., Dottori M. (2013). Effect of temperature on six different developmental landmarks within the pupal stage of the forensically important blowfly *Calliphora vicina* (Robineau-Desvoidy) (Diptera: Calliphoridae). J. Forensic Sci.s.

[39] Richards C.S., Simonsen T.J., Abel R.L., Hall M.J.R., Schwyn D.A., Wicklein M. (2012). Virtual forensic entomology: Improving estimates of minimum post-mortem interval with 3d micro-computed tomography. Forensic Sci. Int..

[40] Tarone A.M., Picard C.J., Spiegelman C., Foran D.R. (2011). Population and temperature effects on *Lucilia sericata* (Diptera: Calliphoridae) body size and minimum development time. J. Med. Entomol..

[41] Warren J.A. (2006). The Development of *Protophormia terraenovae* (Robineau-Desvoidy) (Diptera:Calliphoridae) at Constant and Fluctuating Temperatures. Master of Arts.

[42] Warren J.A., Anderson G.S. (2013). Effect of fluctuating temperatures on the development of a forensically important blow fly, *Protophormia terraenovae* (Diptera: Calliphoridae). Environ. Entomol..

[43] Pickering C.L., Hands J.R., Fullwood L.M., Smith J.A., Baker M.J. (2015). Rapid discrimination of maggots utilising ATR-FTIR spectroscopy. Forensic Sci. Int..

[44] Whitworth T.L. (2006). Keys to the genera and species of blow flies (Diptera: Calliphoridae) of America north of Mexico. Proc. Entomol. Soc. Wash..

[45] Byrd J.H. (2016). Personal communication.

[46] Smith K.G.V. (1986). A Manual of Forensic Entomology.

[47] Kharbouche H., Augsburger M., Cherix D., Sporkert F., Giroud D., Wyss C., Champod C., Mangin P. (2008). Codeine accumulation and elimination in larvae, pupae, and imago of the blowfly *Lucilia sericata* and effects on its development. Int. J. Leg. Med..

[48] Sadler D.W., Fuke C., Court F., Pounder D.J. (1995). Drug accumulation and elimination in *Calliphora vicina* larvae. Forensic Sci. Int..

[49] Gosselin M., Wille S.M.R., del Mar Ramirez Fernandez M., Di Fazio V., Samyn N., De Boeck G., Bourel B. (2011). Entomotoxicology, experimental set-up and interpretation for forensic toxicologists. Forensic Sci. Int..

[50] Bourel B., Tournel G., Hedouin V., Deveaux M., Goff M.L., Gosset D. (2001). Morphine extraction in necrophagous insects remains for determining ante-mortem opiate intoxication. Forensic Sci. Int..

[51] Fleming R.W., Torralba A., Adelson E.H. (2004). Specular reflections and the perception of shape. J. Vis..

[52] Ramsay J.O., Silverman B.W. (2005). Functional Data Analysis.

[53] Makki R., Cinnamon E., Gould A.P. (2014). The development and functions of oenocytes. Annu. Rev. Entomol..

[54] Fan Y., Zurek L., Dykstra M.J., Schal C. (2003). Hydrocarbon synthesis by enzymatically dissociated oenocytes of the abdominal integument of the German cockroach, *Blattella germanica*. Naturwissenschaften.

[55] Blomquist G.J., Blomquist G.J., Bagnères A.-G. (2010). Structure and analysis of insect hydrocarbons. Insect Hydrocarbons Biology, Biochemistry and Chemical Ecology.

[56] Dennell R. (1946). A study of an insect cuticle: The Larval Cuticle of Sarcophaga Falculata Pand. (Diptera). Proc. R. Soc. Lond. Ser. B Biol. Sci..

[57] Dennell R. (1947). A study of an insect cuticle: The formation of the puparium of *Sarcophaga falculata* pand. (Diptera). Proc. R. Soc. Lond. Ser. B Biol. Sci..

[58] Zdarek J., Fraenkel G. (1972). The mechanism of puparium formation in flies. J. Exp. Zool..

[59] Foley W.J., McIlwee A., Lawler I., Aragones L., Woolnough A.P., Berding N. (1998). Ecological applications of near infrared reflectance spectroscopy—A tool for rapid, cost-effective prediction of the composition of plant and animal tissues and aspects of animal performance. Oecologia.

